# Cross-sector Collaboration to Advance Dementia-Friendly Workforce Education in Rural Communities

**DOI:** 10.1093/geroni/igaf122.606

**Published:** 2025-12-31

**Authors:** Jennifer Severance, Janice Knebl, Sara Murphy, Sarah Ross

**Affiliations:** University of North Texas Health Science Center - Fort Worth, TX, Fort Worth, Texas, United States; University of North Texas Health Science Center, Fort Worth, Texas, United States; University of North Texas Health Science Center, Fort Worth, Texas, United States; University of North Texas Health Science Center, Fort Worth, Texas, United States

## Abstract

Limited access to quality dementia care due to workforce challenges is a critical issue, especially in rural communities. Ninety percent of Texas counties are Rural Health areas and 93% are designated as Primary Care Health Professional Shortage Areas. To increase knowledge about dementia in the geriatric care workforce, a Health Resources and Services Administration-funded Geriatric Workforce Enhancement Program in Texas designed collaborative education and training programs aimed towards rural communities. Using a multipronged strategy to engage primary care and long-term care teams across the state, interdisciplinary faculty and community and health care partners combined critical resources to develop and enhance curricula in geriatric best practices using the Institute for Healthcare Improvement’s Age-Friendly Health Systems 4Ms framework. Partners developed an infrastructure to reach rural and underserved communities using learning technologies, social media platforms, and community health educators. An advisory board of older adults and caregivers was assembled to inform curricula development. In the first implementation year, partners incorporated tele-education technologies, experiential learning, and interprofessional activities into learning activities and clinical training environments for health professions students (n = 69), primary care residents (n = 48), health care practitioners (n = 146), and community educators (n = 125). Preliminary trainee feedback demonstrates improved knowledge, confidence and attitudes in providing dementia care and connecting older adults and caregivers to needed social supports. This model of collaboration continues with a goal to further enhance training models, expand organizational capacity to respond to community needs, improve health care for older adults and their caregivers, and build bridges between community and clinical settings.

